# Robust magnon-photon coupling in a planar-geometry hybrid of inverted split-ring resonator and YIG film

**DOI:** 10.1038/s41598-017-12215-8

**Published:** 2017-09-20

**Authors:** Biswanath Bhoi, Bosung Kim, Junhoe Kim, Young-Jun Cho, Sang-Koog Kim

**Affiliations:** National Creative Research Initiative Center for Spin Dynamics and Spin-Wave Devices, Nanospinics Laboratory, Research Institute of Advanced Materials, Department of Materials Science and Engineering, Seoul National University, Seoul, 151-744 Republic of Korea

## Abstract

We experimentally demonstrate strongly enhanced coupling between excited magnons in an Yttrium Iron Garnet (YIG) film and microwave photons in an inverted pattern of split-ring resonator (noted as ISRR). The anti-crossing effects of the ISRR’s photon mode and the YIG’s magnon modes were found from |S_21_|-versus-frequency measurements for different strengths and directions of externally applied magnetic fields. The spin-number-normalized coupling strength (i.e. single spin-photon coupling) ${g}_{{\rm{eff}}}/2\pi \sqrt{N}$ was determined to 0.194 Hz (${g}_{{\rm{eff}}}/2\pi$ = 90 MHz) at 3.7 GHz frequency. Furthermore, we found that additional fine features in the anti-crossing region originate from the excitation of different spin-wave modes (such as the magnetostatic surface and the backward-volume magnetostatic spin-waves) rather than the Kittel-type mode. These spin-wave modes, as coupled with the ISRR mode, modify the anti-crossing effect as well as their coupling strength. An equivalent circuit model very accurately reproduced the observed anti-crossing effect and its coupling strength variation with the magnetic field direction in the planar-geometry ISRR/YIG hybrid system. This work paves the way for the design of new types of high-gain magnon-photon coupling systems in planar geometry.

## Introduction

Information exchange with preserved coherence is essential in quantum-information applications. This explains the long-standing interest in strong coupling between magnetization dynamics and electrodynamics within the field of communication science and technology^1,2^. Recently, due to the advancements in cavity quantum electrodynamics, photon-magnon coupled hybrid systems have been intensively studied both theoretically^2–4^ and experimentally^5–8^. Earlier experimental studies have revealed that coupling between magnons and photons can be readily achieved by inserting a low-damping magnetic material into a high-quality microwave cavity^6–10^. Owing to the strong exchange interaction between individual spins in ferromagnets, uniformly oriented magnetizations can be utilized as a coherent information processing protocol. High spin density (~4.0 × 10^27^ m^−3^) and low-damping (*α*~10^−5^ to 10^−3^) materials, Yttrium Iron Garnet (YIG) for example, give rise to strongly coupled magnon modes^11^, thereby allowing for their coherent quantum effects used for quantum information processing in quantum computing devices.

In most studies on magnon-photon coupling, three-dimensional (3D) structures consisting of a cavity resonator and an YIG sphere have been used in experiments^3–10^ in which the photon and magnon modes were excited by the photon cavity boundary and the spin precession under an external static magnetic field, respectively. Very recently, spin-pumping in combination with inverse spin Hall effect has been employed as a means of detecting magnon-photon coupling in an YIG/Pt hybrid system^12,13^, and has uncovered distinct features not observed in earlier spin-pumping experiments^14–16^. Furthermore, Wang *et al*. demonstrated the magnon Kerr effect in a strongly coupled cavity-magnon system^17^, and Hisatomi *et al*. measured GHz-frequency Faraday rotations of light polarization in a hybrid YIG sphere/3-D cavity system^18^.

Although 3D cavity resonators have been much employed in the study of magnon-photon coupling, planar-geometry resonator-based systems reportedly exhibit higher coupling strengths than those of the 3D cavity resonator-based hybrids^19–24^. Also, the sphere-shaped YIG system^3–10^ is less compatible with the current Complementary Metal-Oxide-Semiconductor (CMOS) platforms incorporated in planar geometry. Therefore, the most recent studies have focused on hybrids composed of YIG (or Py, FeCo alloys) thin film and the split-ring resonator (SRR) in planar geometry^20,22–24^, because such hybrid systems offer an advantage with respect to integration with current on-chip devices^25^.

Furthermore, it is demonstrated that the excitation of higher-order spin-wave modes plays a more significant role in enhancing the coupling strength^9,22^. Although the cavities have a quality factor higher than those of the planar (e.g. SRR or inverted-SRR) structures, their inability to excite higher-order spin-wave modes in magnetic systems^26,27^ does not sufficiently affect the magnon-photon coupling strength in cavity-based systems. To excite the higher-order spin-wave modes, the cavities need to be specially designed^28^, but this always is difficult. This disadvantage of the cavity system is not an issue in stripline-planar resonator-based systems. On the other hand, the planar structure and easy localization of microwave fields both allow for much excitation of the higher-order spin-wave modes, which can also contribute to photon-magnon coupling^22,24^.

In the present study, we investigated magnon-photon coupling at room temperature in a compact planar hybrid system consisting of an inverted SRR pattern (hereafter noted as ISRR) and an YIG thin film. In this ISRR-YIG hybrid, we found strong magnon-photon coupling along with a nearly five times higher gain and a wider frequency band relative to those of the SRR of the same dimensions as the ISRR. The spin-number-normalized coupling strength showed an enhanced value among those reported so far for 3D systems^4,8–10,12,29^ and SRR-based planar hybrids^20–24^. The magnetic-field-direction-dependent photon-magnon coupling is also presented and interpreted using an equivalent circuit model in consideration of spin-wave excitations at specific measurement geometries.

## Results

### Resonator design

**Figure 1:**
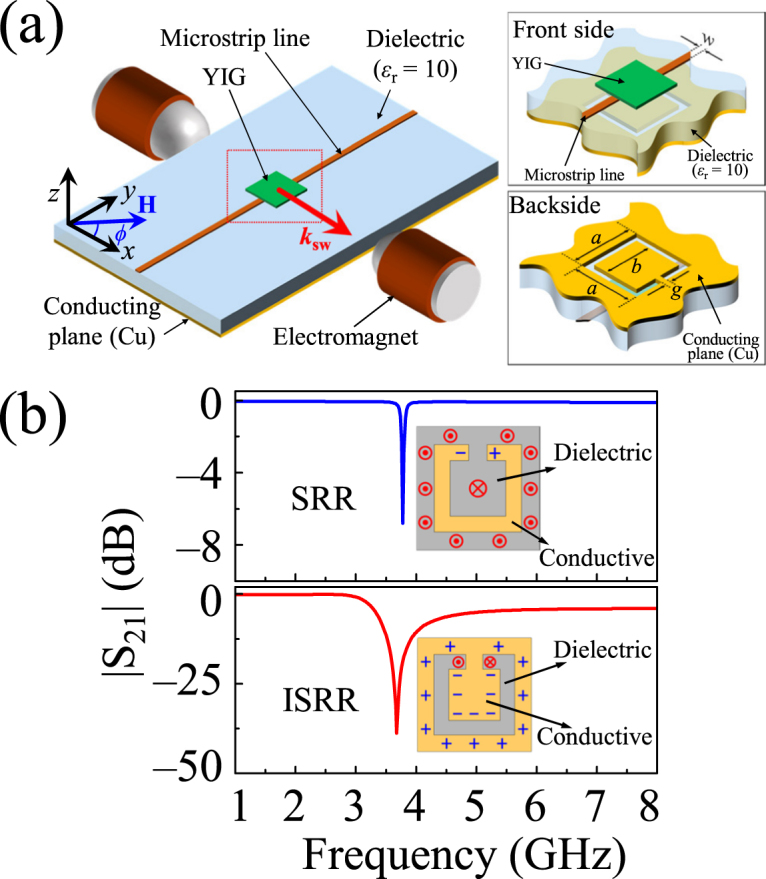
(**a**) Schematic drawing of experimental setup and inverted pattern of the SRR and YIG film (insets). (**b**) Comparison of calculated |S_21_| spectra for the SRR and ISRR structures of same dimensions. The inset shows a schematic of the local electric fields and magnetic fields for each structure. (+) and (−) indicate positive and negative charges, respectively, while (⊗) and (⦿) correspond to the magnetic field entering into and exiting out of the surface, respectively.

The ISRR is an SRR-complementary design. In the ISRR structure, all of the conductive (rings) and dielectric parts of the SRR are replaced with those of a substrate, which, in the present case, was the inverted SRR pattern shown in the insets of Fig. 1a. In the microstrip line arrangement, the electric fields originate from the central strip and terminate perpendicularly on the ground plane. Owing to the presence of the dielectric substrate, the electric fields are tightly concentrated just below the central conductor, and the electric flux density reaches its maximum below the microstrip line^30–32^. To achieve a strong electrodynamic coupling, the ISRR accordingly was designed on the ground plane just below the microstrip line, as shown in the inset of Fig. 1a. When microwave currents flow through the microstrip feeding line (along the *y*-axis), the ISRR is excited by an axial electric field^31,32^. In that state, it behaves as a parallel *LC* resonant circuit, thereby leading to a quasi-static resonant effect^32^.

|S_21_|-versus-frequency spectra for each of the ISRR and SRR structures were calculated using the ANSOFT HFSS software (see Fig. 1b). In the calculation, the shape of the SRR was maintained so as to be consistent with that studied in ref.^20^, while the dimensions were kept the same as those of the ISRR used in this study. The |S_21_| spectra for the ISRR and SRR structures are compared in Fig. 1b. Interestingly, the ISRR shows a much higher gain (40 dB) than does the SRR (7.2 dB), along with a broader bandwidth (156 MHz). Those resonance frequencies are located at the equal frequency of 3.7 GHz, due to the equivalent dimensions of the SRR and ISRR structures.

### Anti-crossing effect between photon and magnon modes

**Figure 2:**
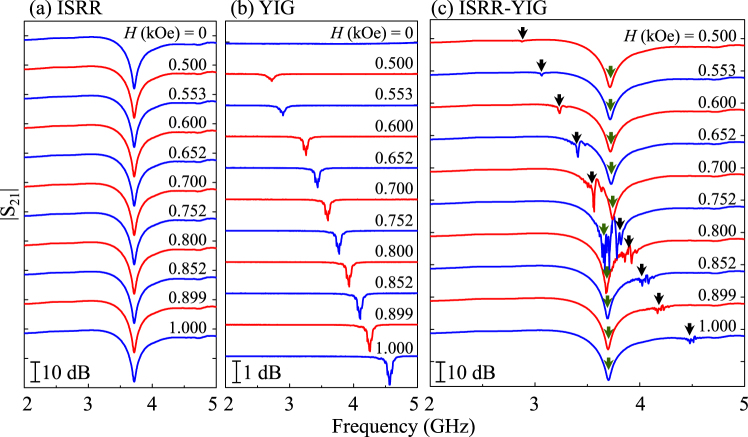
|S_21_| spectra as function of microwave frequency for different field strengths in field direction *ϕ* = 0° for (**a**) ISRR only, (**b**) YIG film only, and (**c**) ISRR-YIG hybrid sample.

We measured (for detail, see Methods) the |S_21_| spectra as a function of the microwave frequency *f* of oscillating currents flowing in the microstrip line for the indicated values of different magnetic-field strengths at an angle *ϕ* = 0°, where *ϕ* is the angle between the magnetic field direction and the *x*-axis. Figure 2a illustrates the |S_21_| spectra measured by applying oscillating currents along the stripline from only the ISRR (without the YIG film). Only the pure photon mode of the ISSR appeared at  *f*_0_ = 3.7 GHz along with a full width at half maximum (FWHM) ∆_FWHM_ ~156 MHz and a quality factor *Q* = *f*_0_/∆_FWHM_ = 25 (see Fig. 2a). The photon mode peak does not move with the field strength. Also, the ferromagnetic resonance (FMR) mode for different *H* values was measured only from the YIG film separately (i.e., without the ISRR) using a co-planar waveguide of 50 Ω, as shown in Fig. 2b. The FMR peak position varies with the magnitude of *H*, because the intrinsic precession motion of the magnetizations varies according to the field strength^26,27^. From Fig. 2a,b, it is evident that the photon mode of the ISRR is ~22 times higher in gain than the magnon modes of the YIG film. However, for the hybrid of the ISRR and the YIG film, there exist two peaks (see Fig. 2c). One peak (marked by black arrows) is weak in gain and very strongly dependent on the applied field strength. Essentially, it continuously shifts towards the higher-frequency side with increasing *H*, and crosses the other peak position (green arrows), thus indicating the FMR mode. The other peak has a relatively high gain and does not much move with increasing *H*, thus indicating the ISRR mode. It is worth noting that when the lower-gain peak approaches the higher-gain peak, the lower-gain peak gradually increases in gain and attains its highest value just as it crosses the other peak, after which its magnitude gradually decreases again with field strength. The small peak is located on the lower-frequency side before it crosses the higher-gain peak, while it is located on the higher-frequency side after it crosses the higher-gain peak. Hereafter, the lower- and higher-frequency peaks are marked as *f*_−_ and *f*_+_, respectively. This evidences that the magnetization-dynamic modes of the YIG film strongly interact with the electrodynamic ISRR mode.

**Figure 3:**
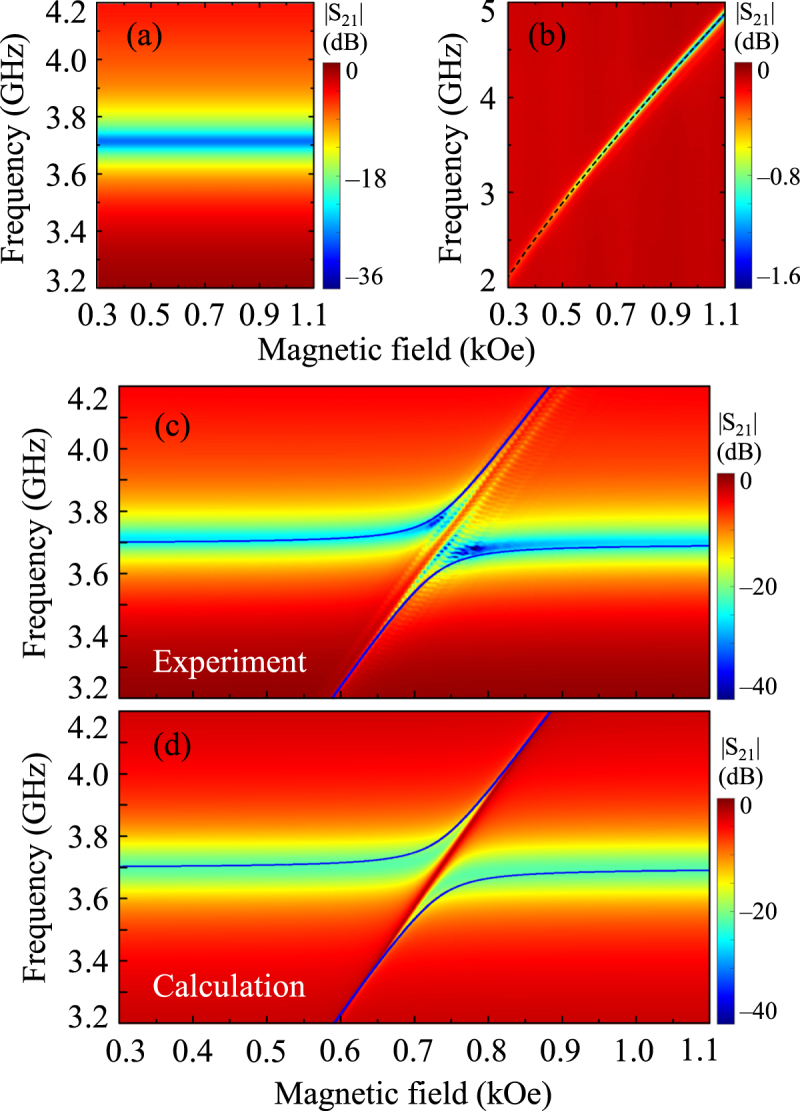
|S_21_| transmission spectra shown in Fig. 2 represented by |S_21_| power on the plane of microwave frequency and magnetic field (*f*-*H* plane), for (**a**) ISRR only, (**b**) YIG film only, and (**c**) ISRR-YIG hybrid sample. (**d**) |S_21_| power on *f*-*H* plane obtained theoretically using equivalent circuit model. The dotted line in (**b**) is the fit to the Kittel’s formula for in-plane position (Eq. (2)).The solid blue lines in (**c**) and (**d**) are the results of fits to Eqs (1–2).

In order to examine the coupling effect shown in Fig. 2, the |S_21_| spectrum powers on the *f*−*H* plane were replotted as indicated in Fig. 3. The |S_21_| spectra measured separately from the ISSR (Fig. 3a) and the YIG film (Fig. 3b) are compared with the results measured for the ISRR-YIG hybrid (Fig. 3c). Far away from the field range of 0.6–0.9 kOe, the resonant frequencies of the hybrid system are very close to that of the single pure ISRR mode, as shown in Fig. 3a. However, being close to that field region, the slope (in the *f*-*H* spectrum in Fig. 3c) becomes similar to the measured FMR frequency-versus-*H* spectrum of the YIG film, as seen in Fig. 3b. From Fig. 3c, it is clear that there exists a strong anti-crossing effect between the ISRR and the FMR modes. Also, in the anti-crossing region (see Fig. 3c), fine-featured lines parallel to the Kittel-type FMR mode are observed, which can be attributed to the magnetostatic spin-wave modes (which will be discussed in later sections).

To analyze the experimentally observed full spectra of the |S_21_| power shown in Figs 2c and 3c, as well as to understand the general features of magnon-photon coupling in the ISRR-YIG hybrid system, we used a circuit model equivalent to the coupled ISRR and YIG system. This model describes the coupling between a resonance mode of the equivalent circuit model (corresponding to the ISRR) and a FMR mode of the YIG film (see Supplementary Material S1). In this model, the microwave currents drive the magnetization precession motion via Ampère’s circuit law, while the dynamic magnetization generates microwave currents through Faraday’s induction law. Using the standard microwave circuit theory, we calculated, for the coupled ISRR and YIG system, the |S_21_| power on the *f-H* plane for *ϕ* = 0° as shown in Fig. 3d, using Eqs (S1–S8) described in Supplementary Materials (for the other angles, see the calculation results shown in Supplementary Fig. S2). The calculation results are in good agreement with the experimentally measured |S_21_| power, except for the fine-featured lines parallel to the Kittel-type FMR mode shown in the corresponding experimental result. Since spin-wave modes are excited at specific angle geometries, we will further discuss the fine features observed in the anti-crossing region.

### Estimation of magnon-photon coupling strength

In order to elucidate the underlying physics of the observed high-gain strong anti-crossing effect from the ISSR-YIG hybrid sample, we considered the degrees of co-operativity of different resonance systems in the field of quantum information. Three different models have been proposed: coupled harmonic oscillators^10,20,21^, dynamic phase correlation^10,29^, and microscopy theory^28,33^. Here, we adapted the harmonic oscillator model, as it can quantitatively describe the experimentally observed modes in coupled systems and also can easily be generalized for a multi-mode coupling system. Based on the coupled harmonic oscillator model, the lower (*f*_−_)- and upper (*f*_+_)-branch frequencies^20,23^ are given by:

1 $${f}_{\pm }=\frac{{f}_{0}+{f}_{{\rm{r}}}}{2}\pm \sqrt{{(\frac{{f}_{0}-{f}_{{\rm{r}}}}{2})}^{2}+{(\frac{{g}_{{\rm{eff}}}}{2\pi })}^{2}},$$

where *f*_0_ and ${f}_{{\rm{r}}}$ are the resonance frequencies of the ISRR mode (photon mode) and the FMR mode in the absence of their coupling, respectively, and *f*_−_ and *f*_+_ represent the lower- and upper-branch frequencies shown in Fig. 2c, respectively. Here the FMR mode represents the precession of uniform magnetizations (Kittel form) without taking into account the spin-wave excitations^11,26,27^. Hence, ${f}_{{\rm{r}}}$ depends on the applied magnetic field strength, as shown in Fig. 3b (fitted well with the Kittel formula, Eq. 2), while *f*_0_ is independent of the applied field strength, as shown in Fig. 3a. The parameter ${g}_{{\rm{eff}}}/2\pi$ in Eq. (1) corresponds to the coupling strength. This coupling strength is measured in the frequency unit that defines the coupling range within which energy can be transferred between the interacting modes of coupled systems^20,21,23^.

The FMR frequency for the in-plane magnetizations without any magnetic anisotropy in the film plane is simply written as^20,26,27^:

2 $${f}_{{\rm{r}}}=\frac{\gamma }{2\pi }\sqrt{H(H+4\pi {M}_{{\rm{S}}}),}$$

where $\gamma /(2\pi )$ is the gyromagnetic ratio (typically 2.8 MHz/Oe for YIG), and $4\pi {M}_{{\rm{S}}}$ is the saturation magnetization (here, 0.172 T for the YIG film). Fitting of Eq. (1) to the experimental data shown in Fig. 3c gave rise to two, higher- and lower-frequency branches, as marked by the solid blue lines. From the fitting, we also obtained ${g}_{{\rm{eff}}}/2\pi$ = 90 MHz (*k* = 0.221) at *f*_0_ =3.7 GHz, where the coupling constant *k* is defined as $k={[2({g}_{{\rm{eff}}}/2\pi )/{f}_{0}]}^{1/2}$, as reported in refs^10,19^. The *k* parameter represents the information exchange rate between the magnon and photon modes during coupling at a particular frequency^10,24^. We compared both values of ${g}_{{\rm{eff}}}/2\pi$ and *k* for the ISRR/YIG hybrid as well as for other systems reported in earlier publications. Table 1 shows those values as obtained from both the planar and the 3D cavity hybrid systems. It is evident that the value of ${g}_{{\rm{eff}}}/2\pi$(or *k*) obtained from the present study is higher than those obtained from the 3D cavity/YIG sphere hybrids^8–13,20,29^, but is lower than those obtained from some of co-axial-^28^ and planar-based hybrid systems^20,24^. Recently, the dependence of the coupling strength on the diameter of YIG spheres with a 3D cavity was demonstrated by Tabuchi *et al*.^4^. Since the coupling strength can be enhanced by either increasing the size of YIG spheres or reducing the microwave cavity size^5^, direct comparison of coupling strengths for a variety of different systems (e.g., 3D or 2D hybrid structures of different dimensions) requires the normalization of the coupling strength by its net spin number *N*. The coupling strength, ${g}_{{\rm{eff}}}/2\pi$, was shown to be proportional to $\sqrt{N}$, as ${g}_{{\rm{eff}}}={g}_{0}\sqrt{N}$, where ${g}_{{\rm{0}}}$ is the coupling strength of a single Bohr magneton to the microwave photon^4^. Moreover, in 3D hybrids, the YIG sphere is usually kept inside the cavity, so that all of the spins present in the YIG sphere contribute to coupling with photons^4,8–12,28,29^. Similarly, in SRR-based planar hybrids, the YIG film is positioned over the SRR, and thus the total spins of only the YIG film that is in direct contact with the SRR, not of its entire volume, contribute to the coupling^20,24^. Therefore, using the equal spin density value for the YIG film used in refs^9,20^, the spin-number-normalized coupling strength (i.e. single spin-photon coupling) for different 3D and planar hybrid systems was estimated by considering, as discussed above, the net spin numbers of only the effective volume of YIG that contributes to coupling. The net spin-number of our sample was estimated to be *N* = 0.21 × 10^18^ according only to the effective volume of the YIG film in contact with the 0.55 mm width microstrip line (i.e., *V* = 3.7 mm × 0.55 mm × 25 *µ*m). This value is one order of magnitude smaller than the value of 7.62 × 10^18^ for the sample reported in ref.^20^, and relatively smaller than the value of 2.2 × 10^18^ for the sample reported in ref.^28^. Thus, in our case, the spin-number-normalized coupling strength was estimated to be ${g}_{{\rm{eff}}}/2\pi \sqrt{N}$ = 0.194 Hz. The corresponding spin-number-normalized coupling constant (${k}_{N}$) was found to be 1.025 × 10^−2^. As indicated in Table 1, the observed value of ${g}_{{\rm{eff}}}/2\pi \sqrt{N}$ for the ISRR-YIG hybrid used in the present study was found to be higher than the values obtained for most of the 3D-^4,8–10,12,13,28,29^ and SRR-based planar hybrids^20–24^. It should be noted that in refs^20,24^, the measurements were performed while keeping the YIG film directly on the SRR; in the present investigation though, the ISRR was not in atomic-scale physical contact with the YIG film and yet still showed a higher value of ${g}_{{\rm{eff}}}/2\pi \sqrt{N}$ than those reported for the other SRRs. This enhancement in the coupling strength of the ISRR/YIG system is mainly due to the compactness of the resonator and the higher gain of the inverted pattern of an SRR. The ISRR/YIG hybrid we studied offers an advantage over the 3D-based and/or the SRR-based hybrids in terms of photon-magnon coupling strength.

**Table 1:** Comparison of coupling strength and other parameters obtained in this work with those reported in literature.

System	*f*_0_[GHz]	$\frac{{{\bf{g}}}_{{\boldsymbol{eff}}}}{{\bf{2}}{\boldsymbol{\pi }}}[{\bf{MHZ}}]$	*k*	$\frac{{{\boldsymbol{g}}}_{{\boldsymbol{eff}}}}{{\bf{2}}{\boldsymbol{\pi }}\sqrt{{\boldsymbol{N}}}}[{\bf{Hz}}]$	*k*_*N*_ × 10^−2^	Reference
YIG film/3-D cavity	10.565	47	0.094	0.031	0.245	4
YIG film/3-D cavity	10.847	65	0.109	0.056	0.323	8
YIG film/3-D cavity	7.9	23	0.076	0.033	0.292	9
YIG sphere/3-D cavity	10.556	31.5	0.077	0.021	0.20	10
YIG film/3-D cavity	10.506	80	0.123	0.069	0.364	12
YIG film/3-D cavity	9.65	24.2	0.081	0.090	0.434	13
YIG sphere/co-axial cavity	3.535	130	0.271	0.087	0.704	28
YIG sphere/3-D cavity	12.375	30.3	0.070	0.020	0.182	29
YIG film/SRR on stripline	3.2	270	0.411	0.097	0.782	20
YIG sphere/SRR on stripline	4.08	65	0.170	0.040	0.445	21
YIG cylinder/SRR stripline	4.75	32.85	0.117	0.004	0.137	22
YIG film/SRR on CPW	10.9	150	0.165	0.120	0.462	24
ISRR/YIG film	3.7	90	0.221	0.194	1.025	Present work

### Fine-featured coupling modes in the anti-crossing region due to spin-wave excitations

**Figure 4:**
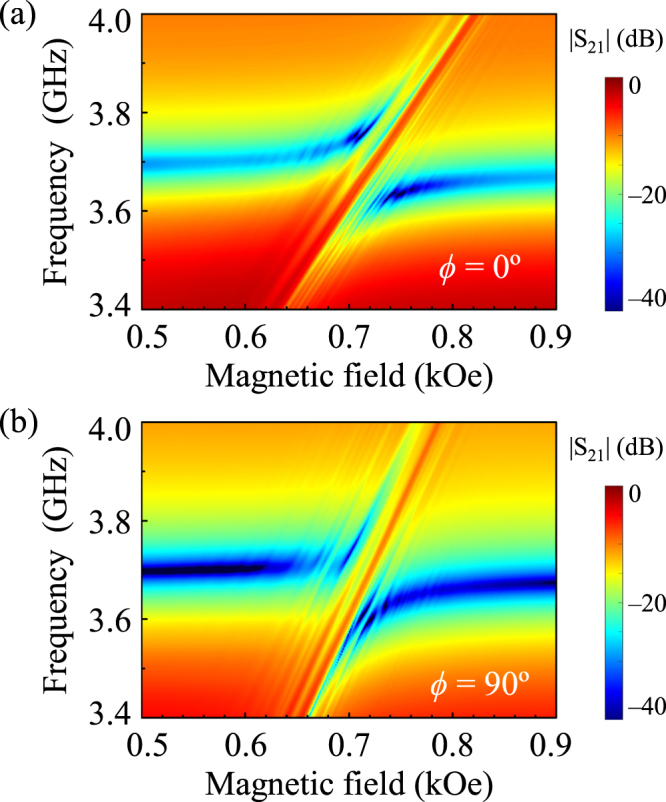
|S_21_| power on *f*-*H* plane measured in very fine field steps of 1 Oe for (**a**) *ϕ* = 0 and (**b**) *ϕ* = 90°.

**Figure 5:**
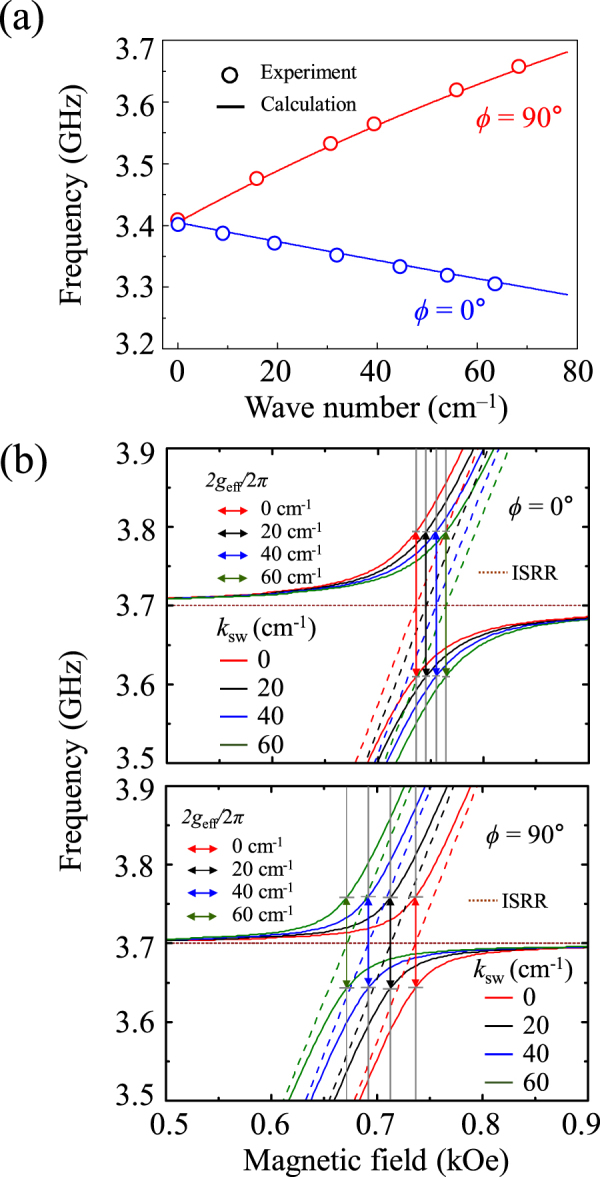
(**a**) Dispersion characteristics for MSSW and BVMSW excitations for bias magnetic field *H* = 0.6 kOe applied at *ϕ* = 0 and 90°. Symbols: experimental data; solid lines: calculated using Eq. (S9). (**b**) Anti-crossing of ISRR and YIG modes as obtained theoretically using equivalent circuit model when *ϕ* = 0 and 90° for *k*_sw_ = 0, 20, 40, and 60 cm^−1^.

It is well known that YIG thin films show three types of magnetostatic spin-waves, namely magnetostatic surface spin-waves (MSSWs), forward volume magnetostatic spin-waves (FVMSWs), and backward volume magnetostatic spin-waves (BVMSWs), the dispersion relations of which distinctly differ from each other^26^. In our measurement geometries (in-plane magnetized film), two types of spin-waves can be excited. One is the MSSWs (at *ϕ* = 90°) that propagate in the film plane in a direction perpendicular to the applied magnetic field. This type is characterized by a positive group velocity, $df/d{k}_{{\rm{sw}}}$ > 0, where *k*_sw_ is the wave number. The other type of spin-waves is the BVMSWs at *ϕ* = 0° that propagate in the same direction as that of the applied magnetic field with a negative group velocity, *df*/*dk*_sw_ < 0^26^. In our experimental geometries, these spin-waves are excited by microwave Oersted fields around the microstrip line and always propagate along the axis (the *x*-axis in Fig. 1a) perpendicular to the microstrip line. The frequency (energy) gaps ∆*f* at *k*_sw_ = 0 for both types of spin-waves are the same as given by the Kittel form. In the YIG film, there exist a large number of *k*_sw_ of the excited spin-waves. Therefore, in order to elucidate the underlying physics of the fine features observed in Fig. 3c, we also carried out fine measurements of the |S_21_| power on the *f-H* plane with fine field steps of 1 Oe for two different field directions, *ϕ* = 0 and 90°, as shown in Fig. 4a,b, respectively. At *ϕ* = 0 and 90°, the BWMSWs and MSSWs, respectively, are excited. The excitation of spin-waves of different wave numbers gives rise to such fine-featured lines parallel to the Kittel type mode (see the many lines in the Kittel-type resonant mode in Fig. 4a,b). Thus, the wave number *k*_sw_ of these spin-waves excited in the YIG film can be estimated directly from their respective resonance frequencies using the modified Kittel Eq. (S9) for *ϕ* = 0 and 90°. To identify the peak positions of the excited spin-waves, we used the |S_21_| spectra measured at *H* = 0.6 kOe for *ϕ* = 0 and 90° (see Supplementary Fig. S3). Figure 5a shows the experimentally observed dispersion characteristics ($f$-versus-*k*_sw_) for *ϕ* = 0 and 90°, in which geometries negative (for BVMSW) and positive (MSSW) slopes, respectively, are clearly identified. Also, considering the BVMSW (at *ϕ* = 0°) and MSSW (at *ϕ* = 90°) excitations, the experimental data are in somewhat good agreement with the theoretical calculations obtained using the modified Kittel equation (see Eq. S9 in Supplementary Material S3).

These excited spin-waves in the YIG film contribute to their magnon coupling with the ISRR resonance mode and thus modify the anti-crossing effect as well as their coupling strengths. It is evident that the fine features parallel to the FMR mode (the slope of the *f-H* spectrum) are due to the excitations of many of the spin-wave modes. To interpret such fine coupling features, we numerically calculated the |S_21_|-versus-*H* spectra at *ϕ* = 0 and 90° for different *k*_sw_ values (e.g., 0, 20, 40 and 60 cm^−1^) using the modified Kittel equation (Eq. (S9)) and Eqs (S1–S8) (for details, see Supplementary Materials). For the theoretical calculation, the coupling constants *k* estimated for *ϕ* = 0 (*k* = 0.221) and 90° (*k* = 0.172) from the fitting of the experimental data to Eq. (1) were used in Eq. (S5). From the calculation, we defined the center position (*H*_cent_) of the anti-crossing region in the magnetic field strength at which the ISRR mode (dotted line) and the Kittel type mode (dashed lines) cross each other, as shown in Fig. 5b. With the increase in *k*_sw_, the BVMSWs (MSSWs) excited at *ϕ* = 0° (90°) result in a shift of the *H*_cent_ toward the higher (lower) field side. The shifting of the center position is larger for *ϕ* = 90° than for *ϕ* = 0°, as clearly seen in Fig. 5b. Since the MSSW (*ϕ* = 90°) mode has a positive dispersion curve^26^, the increase of *k*_sw_ pushes the anti-crossing region to the higher-frequency side with respect to the ISRR mode’s frequency, thus, resulting in the overall shifting of the anti-crossing center towards the lower-field side. On the other hand, since the BVMSW (*ϕ* = 0°) mode has a negative dispersion, the center position of anti-crossing moves to the higher-field side with increasing *k*_sw_.

### Magnetic-field angular dependence of anti-crossing effect

**Figure 6:**
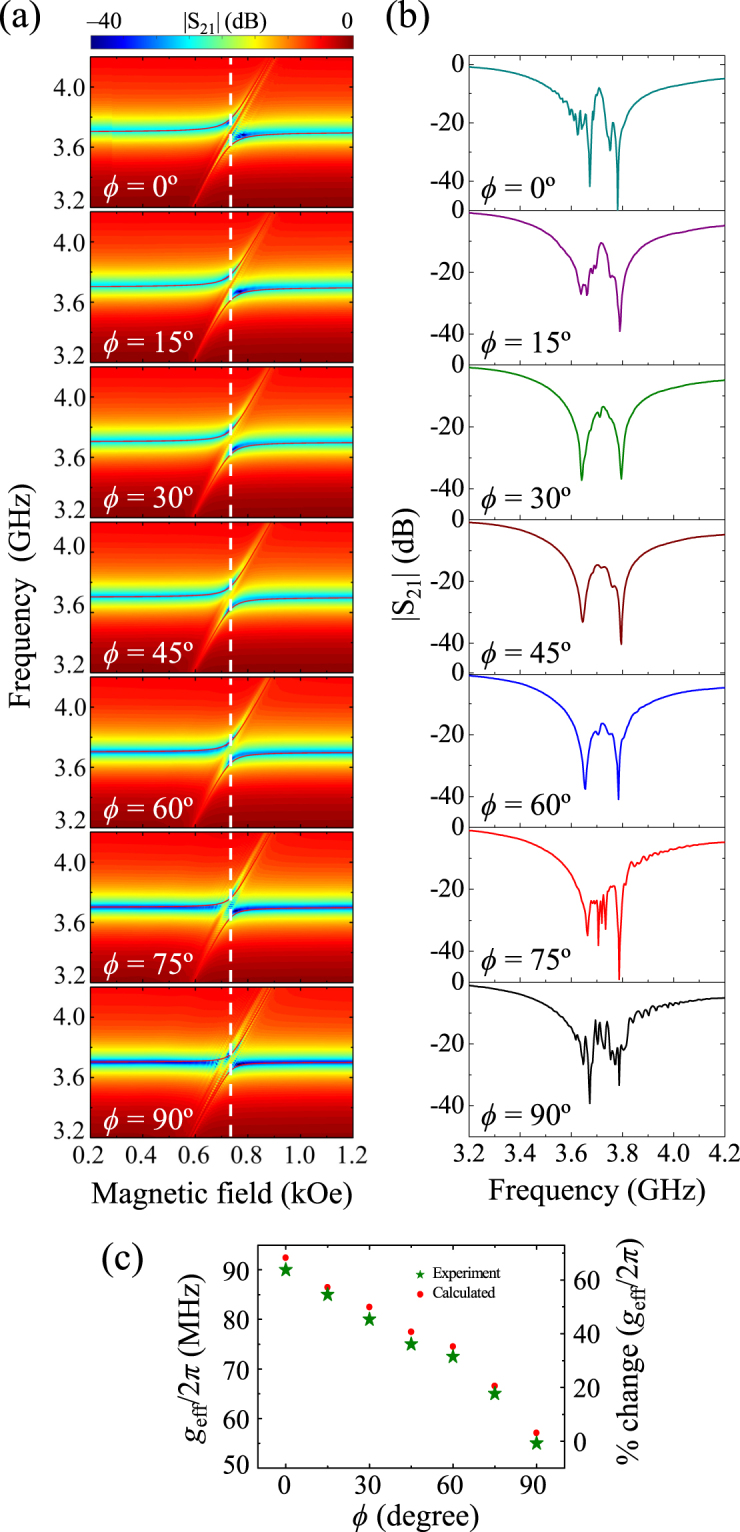
(**a**) |S_21_| power on *f-H* plane as measured for different angles, *ϕ* = 0, 15, 30, 45, 60, 75, and 90°.The solid red lines are the results of fitting to Eqs (1–2) for the coupled oscillator model. (**b**) |S_21_|-versus-frequency spectra for single field of *H* = 0.738 kOe indicated by dashed vertical line in (**a**). (**c**) Variation of ${g}_{{\rm{eff}}}/2\pi$ and percentage of change in ${g}_{{\rm{eff}}}/2\pi$ (for both experiment and calculated results) as a function of angle *ϕ*.

The |S_21_| spectra change remarkably with the field direction. We measured the angular dependence of the photon-magnon coupling by varying the magnetic field direction *ϕ* on the *x-y* plane with respect to the *x*-axis (see Fig. 1a). Figure 6a,b illustrate the experimentally observed |S_21_| power on the *f-H* plane and the |S_21_|-versus-*f* spectra at the specific field strength of *H* = 0.738 kOe, respectively, for the different angles *ϕ* = 0, 15, 30, 45, 60, 75 and 90°. For *ϕ* = 0, 15, 75, and 90°, the extra fine features of the coupling modes were observed in addition to the two fundamental coupling modes (see only the two, fundamental coupling peaks for *ϕ* = 30°) between the ISRR and the YIG film. As the applied magnetic fields deviated from both the *ϕ* = 0 and 90° orientations, the number of fine-feature peaks gradually decreased.

The coupling strengths for different *ϕ* values were estimated by fitting Eqs (1–2) to the experimental data shown in Fig. 6a. The red solid lines for the |S_21_| power on the *f*-*H* plane (see Fig. 6a) are the results of the fitting. Figure 6c shows that the experimental (green symbols) estimation of ${g}_{{\rm{eff}}}/2\pi$ monotonically decreases with the rotation of *ϕ* from 0 to 90°. It is evident too that ${g}_{{\rm{eff}}}/2\pi$ = 90 MHz (*k* = 0.221) at *ϕ* = 0° is reduced to 55 MHz (0.172) at *ϕ* = 90° by 64%. The numerical calculation of ${g}_{{\rm{eff}}}/2\pi$ (red circles) calculated from the equivalent circuit model (shown in Fig. S2) are in good agreement with the experimental result (green stars). At the measurement geometry of *ϕ* = 0°, the BVMSWs are excited, whereas at *ϕ* = 90° the MSSWs are excited. The latter is more localized at the film surface and exponentially decays along the depth of the film. Therefore, the MSSW mode results in less coupling with the ISRR mode than does the BVMSW mode. This leads to the reduction of coupling strength with increasing *ϕ*, as shown in Fig. 6c.

Since the coupling parameters characterize the efficiency of energy transfer between the spin and photonic systems, the above-noted tunable anti-crossing and coupling strength variation along with relatively large gains, as obtained from the YIG/ISRR hybrid system, represents a further step towards the eventual implementation of such novel behaviors of magnetically tunable photon-magnon coupling in real device applications.

### Angular variation of coupling strength and anti-crossing center position

**Figure 7:**
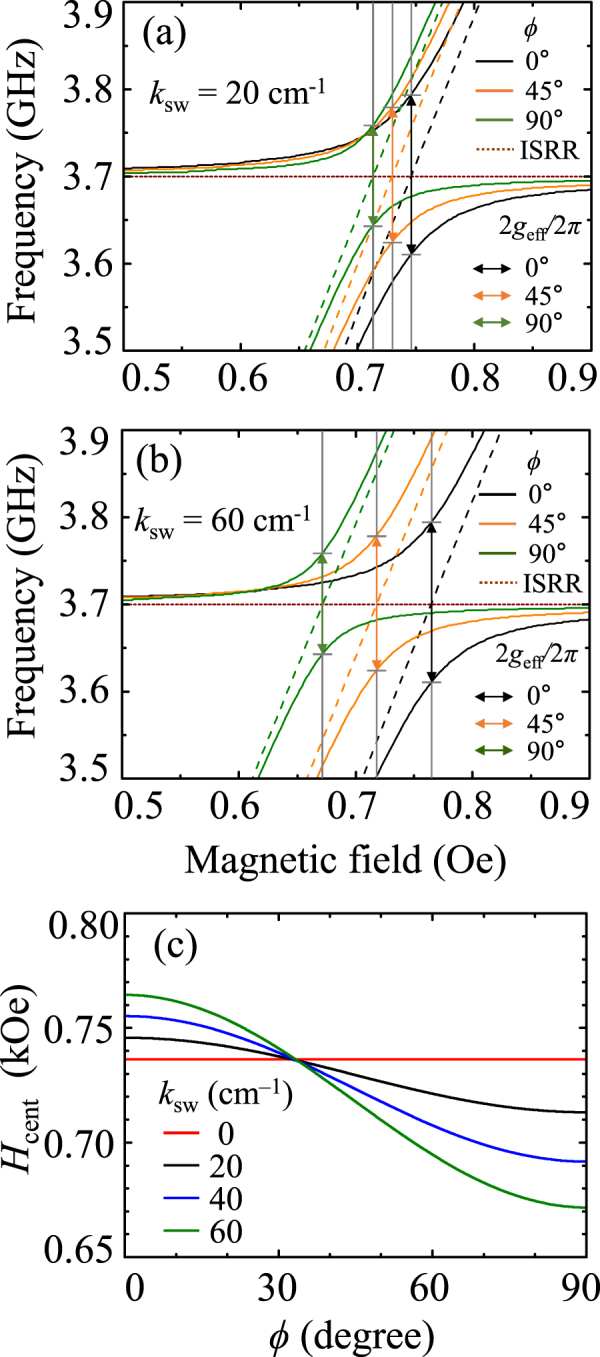
Representation of ${g}_{{\rm{eff}}}/2\pi$ and *H*_cent_ for different values of *ϕ* = 0, 45, and 90° for *k*_sw_ = 20 cm^−1^ in (**a**) and 60 cm^−1^ in (**b**). (**c**) Variation of field center position in anti-crossing region as function of angle *ϕ* for different values of *k*_sw_ = 0, 20, 40, and 60 cm^−1^.

In order to examine the coupling strength variation with the field direction, we further numerically calculated the |S_21_|-versus-*H* spectra for different values of *ϕ* from 0 to 90° for *k*_sw_ = 0, 20, 40, and 60 cm^−1^ based on the *RLC* circuit model (described in detail in Supplementary Material S4). For example, in Fig. 7a,b, we show the |S_21_|-versus- *H* spectra obtained from theoretical calculations for *ϕ* = 0, 45, and 90° for both *k*_sw_ = 20 cm^−1^ and 60 cm^−1^, respectively (for the other angles, see Supplementary Fig. S4). The center position *H*_cent_ of the anti-crossing region and ${g}_{{\rm{eff}}}/2\pi$ evidently varied with *ϕ*. Such angular dependences of *H*_cent_ and ${g}_{{\rm{eff}}}/2\pi$ originate from the coupling of the ISRR mode and its different characteristic spin-wave excitations with varying field direction. The *H*_cent_ versus *ϕ* for different *k*_sw_ values is plotted in Fig. 7c, where *k*_sw_ = 0 corresponds to only the Kittel mode. Characteristic spin-wave excitations in different field directions result in the shifting of *H*_cent_, which effect is larger for higher *k*_sw_ values than for lower ones. Interestingly, we obtained a critical angle of *ϕ* ≈ 33° at which all of the wave numbers have the same center position of anti-crossing; this likely indicates that only the fundamental coupling modes exist. This theoretical calculation is also consistent with the experimental result, which showed, as in Fig. 6, that there exist only fundamental coupling modes at *ϕ* = 30°. Although it is necessary to further study the underlying physics of the presence of such a critical angle, from the above-noted results, it is clear that both *H*_cent_ and ${g}_{{\rm{eff}}}/2\pi$ are controllable with *ϕ*, due to the coupling of characteristic spin-wave excitations with the ISSR mode in different field directions.

## Discussion

From a newly found planar hybrid system that consists of a magnetic insulator (here, YIG film) positioned on top of a microstrip line capacitive-coupled to an inverted pattern of SRR (i.e., the ISRR), we found two novel features. The first is strong photon-magnon coupling (${g}_{{\rm{eff}}}/2\pi$ = 90 MHz) with a spin-number-normalized coupling strength of ${g}_{{\rm{eff}}}/2\pi \sqrt{N}$ = 0.194 Hz. It is also worth mentioning that the magnitude (amplitude) of the |S_21_| signals (40 dB) observed from the ISRR-YIG coupled mode is much higher than those of previously studied hybrid systems^4,8–10,19–24^. This signifies that, by coupling between the photon and magnon modes, higher gains of information carriers can be transferred at a faster rate. The second novel feature is the capacity to increase the photon-magnon coupling strength by 64% simply by changing the magnetic field direction. The 64% enhancement in the coupling strength is much larger than that obtained either by changing the 3D cavity volume or the size of the YIG sphere^4,13^. Our approach more over has the additional merit that it does not require changing of the dimensions of the YIG film or of the ISRR.

In order to interpret our experimental observations and the anti-crossing effect, we numerically reproduced the experimental results using an equivalent circuit model. As this model successfully explains the coupling mechanism between the photon and magnon modes, it can be further extended to multi-mode coupled resonators. Additionally, to achieve the desired value of coupling strength, it is also possible to theoretically predict the values of the circuit parameters for a high-*Q*-value resonator as well as those of the magnetic parameters for a magnetic sample. Recently, there has been a surge of interest in multi-mode magnon-photon coupling. Zhang *et al*. reported the coupling of multiple magnon modes by microwave cavity resonance, having placed multiple YIG spheres into a 3D cavity^33^. Lambert *et al*. demonstrated that the non-uniform magnetostatic modes in a YIG sphere can efficiently be excited by designing a 3D cavity of high *Q* (ref.^28^).

Based on the planar-geometry ISRR-YIG hybrid, different spin-wave modes such as MSSWs and BVMSWs are excited at specific geometries in YIG films by changing the magnetic field direction; this, significantly, provides multi-mode coupling with the ISRR-resonant mode. This multi-mode coupling regime is very interesting in both the fundamental and practical aspects within the fields of signal processing, magnon conversion, and quantum memory. The present sample structure has definite advantages over those used in refs^28,33^, owing to its compact design and high signal strength attained by designing an ISRR structure of a high *Q* factor, which is to say, a structure of decreased *C*_C_ and increased *L*_C_ achieved by tailoring the ISRR shape and dimensions.

Our experimental observations combined with theoretical calculations might provide a useful quantitative tool to explain strongly coupled magnon-photon systems; additionally, they can be extended to multi-mode coupled systems including higher-order interaction terms. Furthermore, the planar hybrid system presented here, promisingly, can be integrated with other planar electronic and optical devices for information transducer applications entailing broad (i.e., MHz, GHz, THz) frequency ranges.

## Methods

### Sample preparation

On the basis of the simulation result for the |S_21_| spectrum of the ISRR, we fabricated the ISRR to dimensions of *a* = 5 mm, *b* = 3.8 mm, and *g* = 0.4 mm by etching out the metallic portion of the ground plane of the microstrip line according to the shape of the SRR (see insets of Fig. 1a), employing photolithography along with the conventional printed circuit board (PCB) technique. The ISRR was implemented on a standard high-frequency laminate (CER-10 RF substrate) of the following material parameters: relative dielectric constant, *ε*_*r*_ = 10; dissipation factor, 0.0012 at 10 GHz; substrate height, 0.64 mm; thickness of copper cladding, 35 *μ*m. Using the App-CAD microstrip line calculator, we set the dimensions of the microstrip line to 0.55 mm width (*w*) and 30 mm length in order to achieve the characteristic 50 Ω impedance. A commercially available epitaxial YIG film of 3.7 mm × 3.7 mm × 25 *μ*m, grown on a Gadolinium Gallium Garnet (GGG) substrate by liquid phase epitaxy was then placed on top of the microstrip line on the front side of the sample (see Fig. 1a). The FMR measurement performed independently for YIG film (shown in Figs 2b and 3b) yielded the following magnetic parameters: $\gamma /2\pi$ = 2.78 MHz/Oe and $4\pi {M}_{S}$ = 0.172 T, and α = 3.2 × 10^−4^. For the measurements of the ISRR-YIG hybrid, microwave connectors (coaxial to microstrip) were soldered at both ends of the microstrip line.

### |S_21_|measurements

To measure the |S_21_| spectra, we used a calibrated two-port vector network analyzer (VNA, Agilent PNA series E8362C). The input and output of the microstrip feeding line were connected to the ports of the VNA. Static magnetic fields were applied (using an electromagnet) on the plane of the YIG film at different angles *ϕ* with respect to the *x*-axis perpendicular to the microstrip line (see Fig. 1a). During the |S_21_| measurements, we varied both the microwave frequency *f* of oscillating currents flowing in the microstrip line and the static field strength *H* for several specific angles *ϕ* (every 15° from 0 to 90°).

## Electronic supplementary material

Supplementary Information
