# Supplementary material for: Robust magnon-photon coupling in a planar-geometry hybrid of inverted split-ring resonator and YIG film
Source: Sci Rep. 2017 Sep 20;7:11930. doi: 10.1038/s41598-017-12215-8 (PMC5607386; doi:10.1038/s41598-017-12215-8)
Supplement: Supplementary file 1 — Supplementary Information [file 41598_2017_12215_MOESM1_ESM.pdf]

SUPPLEMENTARY MATERIALS

Robust magnon-photon coupling in a planar-geometry hybrid of inverted split-ring resonator and YIG film

Biswanath Bhoi, Bosung Kim, Junhoe Kim, Young-Jun Cho and Sang-Koog Kim[a)]

National Creative Research Initiative Center for Spin Dynamics and Spin-Wave Devices, Nanospinics Laboratory, Research Institute of Advanced Materials, Department of Materials Science and Engineering, Seoul National University, Seoul 151-744, Republic of Korea

[a)] Correspondence and requests for materials should be addressed to S.-K. K (sangkoog@snu.ac.kr).

S1. RLC circuit model

In order to obtain physical insight into the experimentally observed $|S_{21}|$ spectra shown in Figs. 2 and 3, we theoretically calculated the corresponding $|S_{21}|$ spectra using a circuit model equivalent to the ISRR-YIG coupling, as described below. Since the ISRR has much smaller dimensions (5 mm) than the wavelengths (~30 cm at 1 GHz) of electromagnetic waves, we took into account the ISRR as a series of *LC* circuits with lumped inductance L_C and capacitance C_C. The equivalent circuit model for the ISRR with the microstrip line along with their coupling is provided in Fig. S1a. The loss in this system originates mainly from the ohmic loss in the metallic material of the stripline and the ground plane; thus it can be described by the equivalent resistance R_0. The impedance of the *LC* circuit is:

$$Z = R_0 + iX_0, \tag{S1}$$

where X_0 is the reactance as given by:

$$X_0 = \omega L_0 - \frac{1}{\omega C_0}, \tag{S2}$$

with $L_0 = L + L_C$, $1/C_0 = 1/C + 1/C_C$, and $\omega = 2\pi f_0$, where f_0 is the resonance frequency of the pure ISRR under the $X_0 = 0$ condition, and thus can be expressed as:

$$2\pi f_0 = \frac{1}{\sqrt{L_0 C_0}}. \tag{S3}$$

The conductance Y_0 for the given system can be written as:

$$Y_0 = \frac{1}{R_0 + iX_0}. \tag{S4}$$

For the ISRR-YIG hybrid under applied magnetic fields, we need to introduce an additional impedance (Z_r) to the *LC* circuit (shown in Fig. S1b) due to its coupling. Thus, the impedance

of the coupled circuit equivalent to the ISRR-YIG hybrid is given by $R_0 + iX_0 + Z_{\mathrm{r}}$. Recently, Harder, *et al.* derived an explicit form of Z_{r} for a 3-D YIG/cavity coupled system[S1]:

$$Z_{\mathrm{r}} = \frac{-2\pi f_{\mathrm{m}} K^2 L_0 f}{f - f_{\mathrm{r}} + i\alpha f}, \tag{S5}$$

where $K = k^2 \left(f_0 / 2 f_{\mathrm{m}} \right)^{1/2}$, $2\pi f_{\mathrm{m}} = \gamma 4\pi \mathrm{M_S}$, α is the Gilbert damping constant for the YIG film, *k* is the coupling constant estimated from experimental results, and f_{r} is the resonance frequency of the magnon modes, as given by the Kittel Eq. (2) for no spin-wave excitations as well as the modified Kittel's Eq. (S9) for spin-wave excitations. The resonance condition for the whole sequence of the equivalent elements connected in series is $X_0 + \mathrm{Im}(Z_{\mathrm{r}})$ = 0, or equivalently, it is given by the frequency position of the maximum value of the total conductance:

$$Y = \frac{1}{R_0 + iX_0 + Z_{\mathrm{r}}}. \tag{S6}$$

For the sake of simplicity, we first obtained the numerical calculation for ϕ = 30°, at which only two fundamental coupling modes were observed, as shown in Fig. 6b. These two resonance peaks of the coupled system are noted as f_- and f_+ in the anti-crossing region [as reflected in Eq. (1)]. In the theoretical calculation, we followed the procedure used in Ref. S2. Initially, by taking C_0 = 2.0 pF obtained from the SRR structure of a similar shape as that used in Ref. S2, L_0 could be obtained from Eq. (S3) by inserting f_0 = 3.7 GHz experimentally observed from only the ISRR. This extracted value of L_0 allowed us to determine R_0 as given by:

$$R_0 = \omega L_0 / Q, \tag{S7}$$

with the quality factor of Q = 25 experimentally observed from the ISRR. Lastly, with the calculated values of L_0, C_0 and R_0 for the ISRR together with the value of Z_r from Eq. (S5), we could determine the f_- and f_+ values for ϕ = 30° according to $X_0 + \mathrm{Im}(Z_r) = 0$.

Once Y is found from Eq. (S6), the |S21| value can be calculated using the expression[S2]

$$|S_{21}| = 1 - K_C Y, \tag{S8}$$

where K_C is the capacitive coupling coefficient between the ISRR and the YIG-loaded feeding line. In order to obtain the theoretical values of |S21| on the *H-f* plane, the aforementioned parameters are first required. We fitted to the experimental data (Fig. 6a for ϕ = 30°) using Eqs. (S1)-(S8) with the initial value C_0 = 2.0 pF. Then, we iteratively performed the aforementioned procedures until the f_- and f_+ values were equal to the experimental ones for ϕ = 30° at H = 0.738 kOe. The results are C_0 = 2.2 pF, L_0 = 0.32 nH, and R_0 = 5.0 Ω. K_C was found from Eq. (S8) to be 5, using the experimentally measured |S21| spectra for ϕ = 30° at H = 0. These quantities we obtained are quite reasonable, and are comparable to the values usually obtained from the SRR structure of mm dimensions[S2]. Therefore, we followed the same procedure to theoretically calculate the |S21| power on the *f-H* plane for the different angles ϕ = 0, 15, 30, 45, 60, 75, and 90°, as shown in Fig. S2. The theoretical calculations were all in good agreement with the experimental data (shown in Fig. 6a), except for additional fine features in the anti-crossing region.

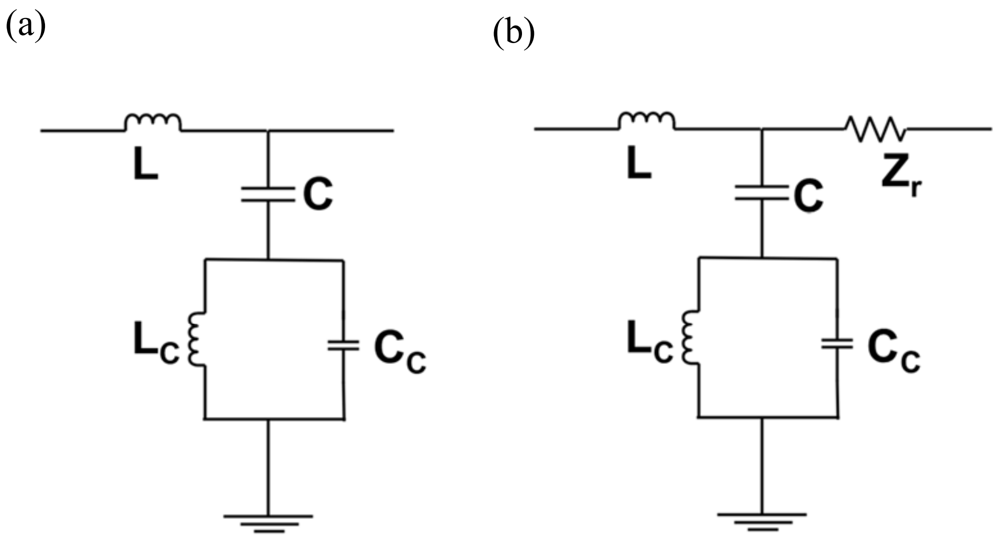

Fig. S1. (a) Circuit model equivalent to only ISRR and (b) *RLC* circuit model for ISRR-YIG hybrid.

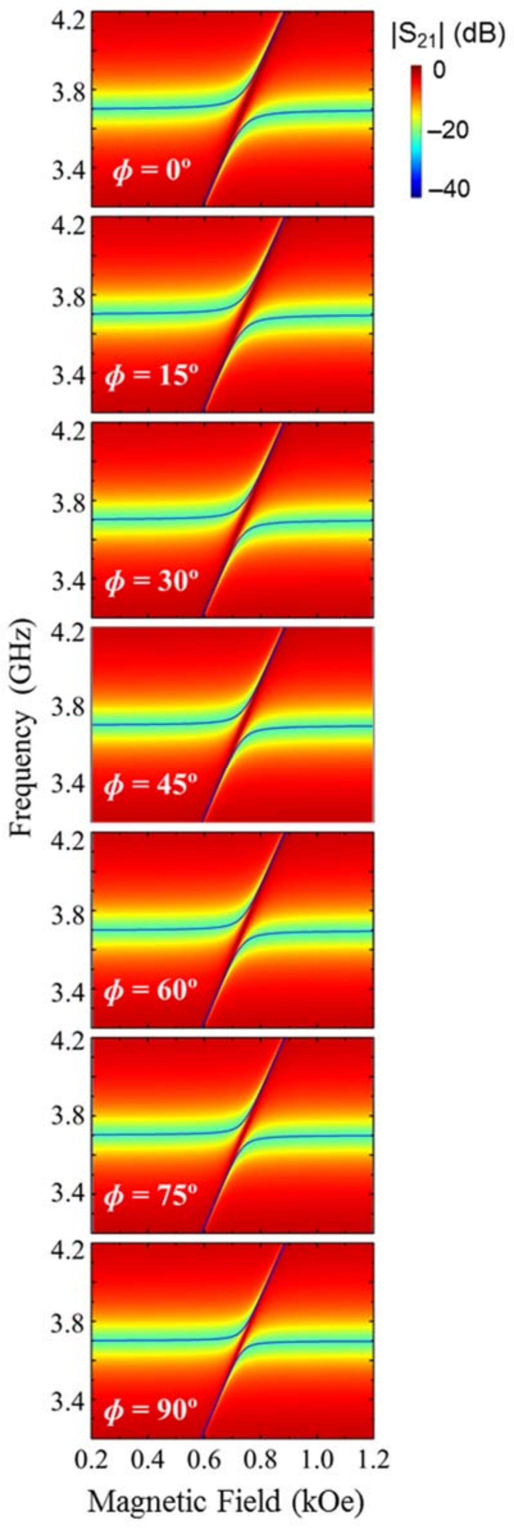

Fig. S2. $|S_{21}|$ spectra on *f*-*H* plane as obtained from theoretical calculations based on equivalent circuit model (considering only fundamental coupling modes) for ϕ = 0, 15, 30, 45, 60, 75, and 90°. The solid blue lines are the results of the fitting of Eqs. (1) – (2) to the theoretically obtained $|S_{21}|$ spectra.

S2. Estimation of wave vectors for ϕ = 0 and 90°

The excitation of spin-waves in the YIG film leads to the generation of multiple resonance peaks in the $|S_{21}|$ spectra, as shown Fig. S3. The strongest peak is the fundamental FMR mode of $k_{sw} \approx 0$. The other, subsidiary peaks are considered as spin-wave modes of different k_{sw} values. Those k_{sw} values were estimated directly by inserting their respective frequency values of local minima in Fig. S3 into the modified Kittel Eq. (S9). Note that the small peak present very close to the fundamental peak (on the low-frequency side in Figs. S3a and Fig.S3b) was not used for the calculation of the k_{sw} values.

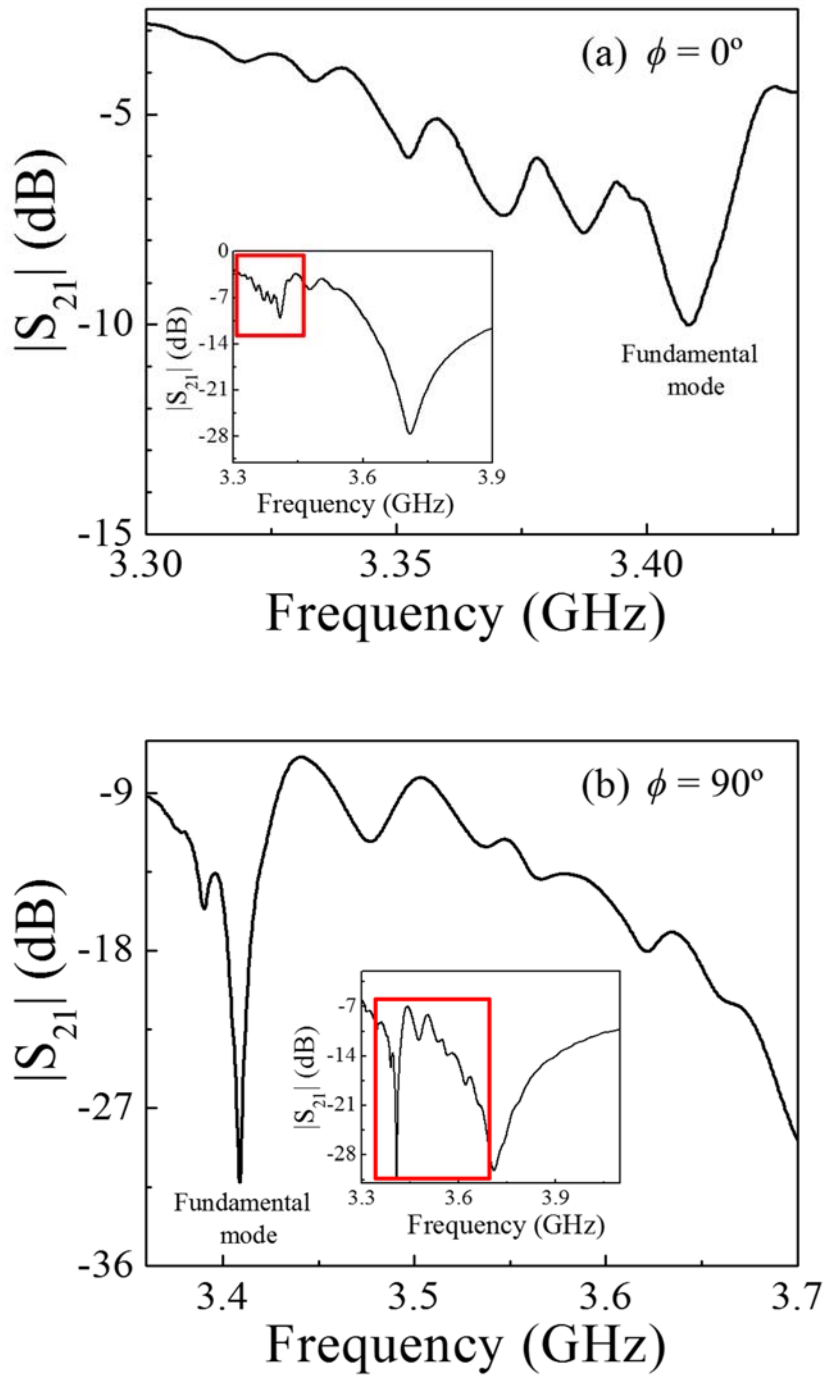

Fig. S3. Magnified $|S_{21}|$ spectra around the fundamental coupling mode, as measured at H = 0.6 kOe for both ϕ = 0 and 90°. The insets show the corresponding full-range $|S_{21}|$ spectra.

S3. Dispersion relations taking into account spin-wave excitations

In the theoretical calculations of |S21|, we used the modified Kittel's equation considering spin-wave excitations[S3]:

$$f_{\mathrm{r}} = \frac{\gamma}{2\pi}\sqrt{\left(H + A_{\mathrm{ex}}k_{\mathrm{sw}}^{2}\right)\left(H + A_{\mathrm{ex}}k_{\mathrm{sw}}^{2} + 4\pi \mathrm{M_S} F_0\left(k_{\mathrm{sw}},\phi,d\right)\right)}, \qquad \text{(S9a)}$$

where A_{ex} is the exchange stiffness (5.3×10^{-13} G·m^2 for the YIG film)[S4], $F_0\left(k_{\mathrm{sw}},\phi,d\right)$ is the matrix element of the dipole-dipole interaction as well as a function of the film thickness *d* and the in-plane magnetic field angle ϕ. The explicit form of $F_0\left(k_{\mathrm{sw}},\phi,d\right)$ is given as[S3]:

$$F_0\left(k_{\mathrm{sw}},\phi,d\right) = 1 + P_0\left(k_{\mathrm{sw}}\right)\left[1 - P_0\left(k_{\mathrm{sw}}\right)\right]\frac{4\pi \mathrm{M_S}}{H + A_{\mathrm{ex}}k_{\mathrm{sw}}^{2}}\sin^2\phi - P_0\left(k_{\mathrm{sw}}\right)\cos^2\phi, \qquad \text{(S9b)}$$

with $P_0\left(k_{\mathrm{sw}}\right) = \left(1 - \frac{1 - e^{-k_{\mathrm{sw}}d}}{k_{\mathrm{sw}}d}\right)$.

Since two different types of spin-waves were excited at ϕ = 0° (BVMSW) and 90° (MSSW), by inserting each of ϕ = 0° and 90°, the dispersion curves for BVMSW and MSSW, respectively, are rewritten as[S5]:

$$f_{\mathrm{BVMSW}} = \frac{\gamma}{2\pi}\sqrt{H\left(H + 4\pi \mathrm{M_S}\frac{1 - e^{-k_{sw}d}}{k_{\mathrm{sw}}d}\right)}, \qquad \text{(S10a)}$$

$$f_{\mathrm{MSSW}} = \frac{\gamma}{2\pi}\sqrt{\left(H + \frac{4\pi \mathrm{M_S}}{2}\right)^2 - \left(\frac{4\pi \mathrm{M_S}}{2}\right)^2 e^{-2k_{sw}d}}. \qquad \text{(S10b)}$$

S4. Theoretical calculation of coupling strength and center position of anti-crossing as function of magnetic field direction

We theoretically calculated the $|S_{21}|$ spectra using Eqs. (S1)-(S9) for each of ϕ = 0, 15, 30, 45, 60, 75, and 90° and for k_{sw} = 0, 20, 40, and 60 cm^{-1}. For all of the curves, the center field position (H_{cent}) in the anti-crossing region was defined as the point where the ISRR mode and spin-wave mode intersect. Figure S4a shows the $|S_{21}|$ spectra for each ϕ at different k_{sw} values. For ϕ = 30°, only the fundamental coupling modes between the ISRR mode and the Kittel FMR mode appear, while for the other angles, additional coupling modes appear. The $|S_{21}|$ spectra for different k_{sw} = 0, 20, 40, and 60 cm^{-1} at each of ϕ = 0, 15, 30, 45, 60, 75, and 90° are shown in Fig. S4b. These indicate a large shifting of H_{cent} in the anti-crossing region.

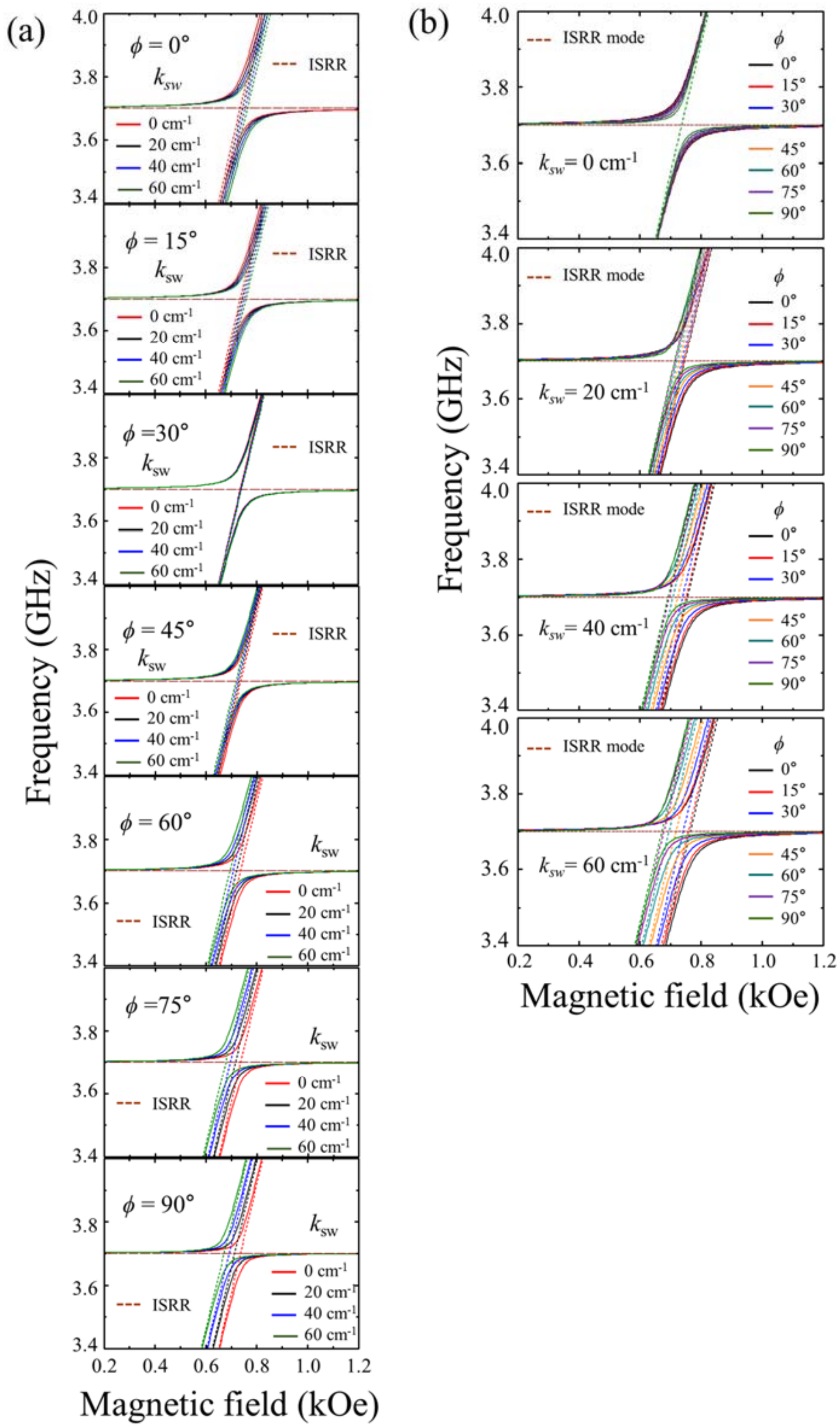

Fig. S4. Theoretical calculation of anti-crossing of the ISRR and YIG modes: (a) for each of ϕ = 0, 15, 30, 45, 60, 75, and 90°, we varied k_{sw} = 0, 20, 40, and 60 cm^{-1}; (b) for each of k_{sw} = 0, 20, 40, and 60 cm^{-1}, we varied ϕ = 0, 15, 30, 45, 60, 75, and 90°.

S5. Electric and magnetic field distribution in ISRR

In the microstrip line arrangement, the electric fields originate from the central strip and terminate perpendicularly on the ground plane. When microwave currents flow through the microstrip feeding line the ISRR is excited by an axial electric field. The electric and magnetic field distribution in ISRR is shown in Fig S5.

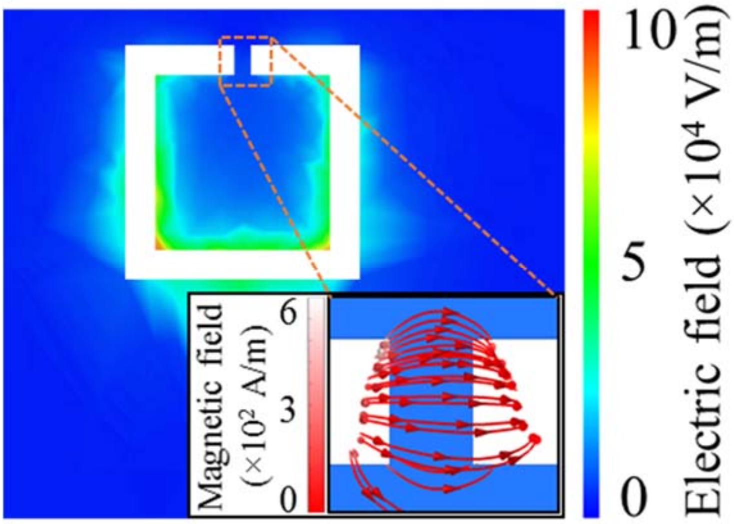

Fig. S5. Distribution of local electric fields in the ISRR along with the distribution of local magnetic field perpendicular to the metallic surface (inset).

Supplementary References

S1. Harder, M., Bai, L., Match, C., Sirker, J., and Hu, C. Study of the cavity magnon-polariton transmission line shape. *Science China Physics, Mechanics &Astronomy*, **59,** 117511 (2016).

S2. Bhoi, B. *et al.* Study of photon-magnon coupling in a YIG-film split-ring resonant system. *J. App. Phys*. **116,** 243906 (2014).

S3. Kalinikos. B. A., and Slavin. A. N. Theory of dipole-exchange spin wave spectrum for ferromagnetic films with mixed exchange boundary conditions. *J. Phys. C: Solid State* **19,** 7013 (1986).

S4. Klingler, S. *et al.* Measurements of the exchange stiffness of YIG films using broadband ferromagnetic resonance techniques. *J. Phys. D: Appl. Phys*. **48,** 015001 (2015).

S5. Serga, A., Chumak, A. V., and Hillebrands, B. YIG magnonics. *Phys. D: Appl. Phys.* **43,** 264002 (2010).
